# Dephosphorylation of CDK9 by protein phosphatase 2A and protein phosphatase-1 in Tat-activated HIV-1 transcription

**DOI:** 10.1186/1742-4690-2-47

**Published:** 2005-07-27

**Authors:** Tatyana Ammosova, Kareem Washington, Zufan Debebe, John Brady, Sergei Nekhai

**Affiliations:** 1 Center for Sickle Cell Disease, Howard University, 2121 Georgia Ave., N.W. Washington DC 20059, USA; 2 Department of Biochemistry and Molecular Biology, Howard University College of Medicine, 520 W Street N.W., Washington, DC 20059, USA; 3 Virus Tumor Biology Section, LRBGE, National Cancer Institute, Bethesda, MD 20892, USA

## Abstract

**Background:**

HIV-1 Tat protein recruits human positive transcription elongation factor P-TEFb, consisting of CDK9 and cyclin T1, to HIV-1 transactivation response (TAR) RNA. CDK9 is maintained in dephosphorylated state by TFIIH and undergo phosphorylation upon the dissociation of TFIIH. Thus, dephosphorylation of CDK9 prior to its association with HIV-1 preinitiation complex might be important for HIV-1 transcription. Others and we previously showed that protein phosphatase-2A and protein phosphatase-1 regulates HIV-1 transcription. In the present study we analyze relative contribution of PP2A and PP1 to dephosphorylation of CDK9 and to HIV-1 transcription *in vitro *and *in vivo*.

**Results:**

*In vitro*, PP2A but not PP1 dephosphorylated autophosphorylated CDK9 and reduced complex formation between P-TEFb, Tat and TAR RNA. Inhibition of PP2A by okadaic acid inhibited basal as well as Tat-induced HIV-1 transcription whereas inhibition of PP1 by recombinant nuclear inhibitor of PP1 (NIPP1) inhibited only Tat-induced transcription *in vitro*. In cultured cells, low concentration of okadaic acid, inhibitory for PP2A, only mildly inhibited Tat-induced HIV-1 transcription. In contrast Tat-mediated HIV-1 transcription was strongly inhibited by expression of NIPP1. Okadaic acid induced phosphorylation of endogenous as well transiently expressed CDK9, but this induction was not seen in the cells expressing NIPP1. Also the okadaic acid did not induce phosphorylation of CDK9 with mutation of Thr 186 or with mutations in Ser-329, Thr-330, Thr-333, Ser-334, Ser-347, Thr-350, Ser-353, and Thr-354 residues involved in autophosphorylation of CDK9.

**Conclusion:**

Our results indicate that although PP2A dephosphorylates autophosphorylated CDK9 *in vitro*, in cultured cells PP1 is likely to dephosphorylate CDK9 and contribute to the regulation of activated HIV-1 transcription.

## Background

Transcription of human immunodeficiency virus (HIV-1) is activated by viral Tat protein which binds to a transactivation response (TAR) RNA [1-4]. In cell-free transcription assays Tat exclusively induces elongation of transcription [5,6]. In contrast, Tat induces initiation of transcription from the integrated HIV-1 promoter in the cells [7-9]. In an early study by Jeang and Berkhout, self-cleaving ribozymes introduced into TAR RNA inhibited Tat transactivation when TAR RNA was cleaved quickly, but not when the cleavage was delayed, indicating that the initial contact between Tat and TAR RNA rather than RNAPII pausing was the rate limiting step in Tat transactivation [9]. Recently Green and coworkers showed that Tat stimulates formation of transcription complex containing TATA-box-binding protein (TBP) but not TBP-associated factors (TAFs), thus indicating that Tat may enhance initiation of transcription [7]. This latter finding apparently agrees with the early observation by Kashanchi and colleagues that Tat binds directly to the TBP-containing basal transcription factor TFIID [10]. Tat activates HIV-1 transcription by recruiting transcriptional co-activators that include Positive Transcription Elongation Factor b (P-TEFb), containing CDK9/cyclin T1, an RNA polymerase II C-terminal domain kinase [6,11,12] and histone acetyl transferases [13-15]. Whereas P-TEFb induces HIV-1 transcription from non-integrated HIV-1 template [6,11,12], histone acetyl transferases allow induction of integrated HIV-1 provirus [13-15]. Cyclin T1 interacts with the loop of TAR RNA and with Tat through a critically conserved cysteine; the mutation of which in rodent cells renders Tat transactivation inefficient [16,17]. *In vitro *association of P-TEFb with Tat and TAR RNA is enhanced when CDK9 is autophosphorylated [18]. We previously showed that *in vitro*, unphosphorylated CDK9 associates with the preinitiation complex and its phosphorylation is directly inhibited by TFIIH [19]. Upon dissociation of TFIIH during elongation of transcription, CDK9 undergoes phosphorylation that is induced by Tat [19]. Thus, it appears that CDK9 might need to be dephosphorylated prior to its association with the transcription initiation complex. Previously, two serine-threonine phosphatases, protein phosphatase 2A (PP2A) and protein phosphates-1 (PP1) were implicated in the regulation of HIV-1 transcription. PP2A and PP1 are a general phosphatases that belong to the PPP-family of protein phosphatases with predominant nuclear localization [20]. Nuclear PP2A and PP1 consist of a constant catalytic subunit and a variable regulatory subunits that determines the localization, activity and substrate-specificity of the phosphatase [20]. Protein phosphatase 2A (PP2A) positively regulates HIV-1 transcription as deregulation of cellular enzymatic activity of PP2A inhibited Tat-induced HIV-1 transcription [21,22]. Expression of the catalytic subunit of PP2A enhanced activation of HIV-1 promoter by phorbol myristate acetate (PMA), whereas inhibition of PP2A by okadaic acid and by fostriecin prevented activation of HIV-1 promoter [22]. One of the major nuclear subunits of PP1 is Nuclear Inhibitor of PP1 (NIPP1) that binds to the catalytic subunit of PP1 and form an inactive holoenzyme complex which can be activated by phosphorylation of NIPP1 [23,24]. By using NIPP1 to inhibit nuclear PP1, we have demonstrated that protein phosphatase-1 (PP1) is a positive regulator of HIV-1 transcription *in vitro *[25] and *in vivo *[26]. We hypothesized that positive effect on HIV-1 transcription observed by either PP1 or PP2A could be a result of dephosphorylation of CDK9, which would increase the amount of active P-TEFb available for recruitment to the HIV-1 promoter. In the present paper we performed a comparative analysis of CDK9 dephosphorylation by PP1 and PP2A *in vitro*. Autophosphorylated CDK9/cyclin T1 was subjected to dephosphorylation by PP2A and PP1. Also we analyzed the effect of dephosphorylation of CDK9 by PP2A or PP1 on the complex formation between Tat, TAR RNA and CDK9/cyclin T1. Analysis of the effect of PP2A inhibition on HIV-1 transcription *in vitro *was carried out using okadaic acid, which inhibits PP2A at low concentration. To inhibit PP1 in HIV-1 transcription *in vitro*, we used recombinant NIPP1 protein. In cultured cell, okadaic acid was used to induce phosphorylation of CDK9, and the cells stably expressing central domain of NIPP1 were used to determine whether the okadaic acid induced phosphorylation was a PP1-dependent effect. Finally, we analyzed phosphorylation of CDK9 with mutations in the Thr 186 or with mutations in Ser-329, Thr-330, Thr-333, Ser-334, Ser-347, Thr-350, Ser-353, and Thr-354 residues involved in autophosphorylation of CDK9. Our results indicate that while PP2A dephosphorylates CDK9 *in vitro *and it is PP1 that dephosphorylates CDK9 *in vivo*, and thus might have a regulatory role in HIV-1 transcription.

## Results

### PP2A dephosphorylates CDK9 in vitro

**Figure 1:**
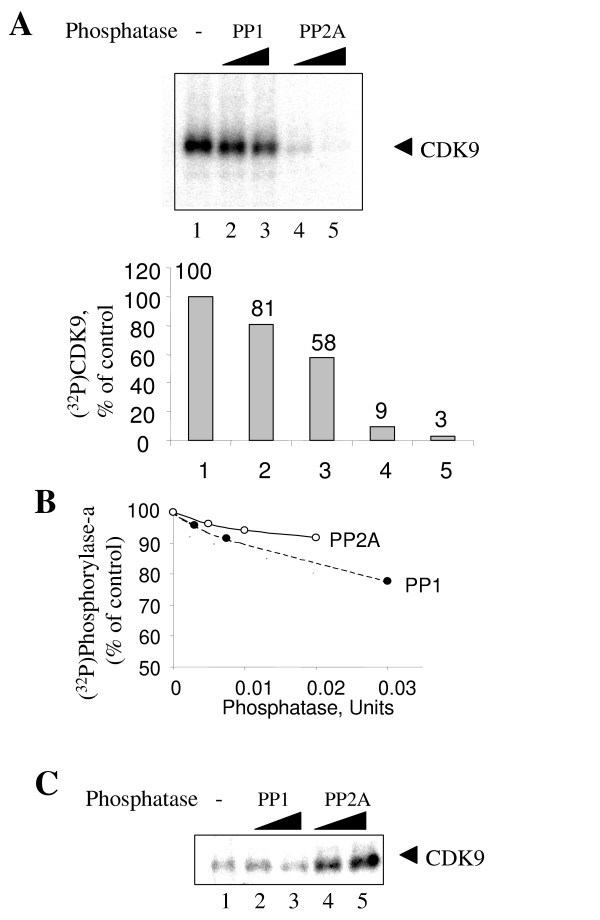
**PP2A dephosphorylates CDK9 *in vitro***. ***A***, Dephosphorylation of CDK9 by PP2A and PP1. Recombinant CDK9/cyclin T1 was incubated with γ-(^32^P) ATP to allow autophosphorylation (lane 1). The kinase activity was blocked by 7 mM EDTA and CDK9 was used as a substrate for PP1 (lanes 2 and 3) or PP2A (lanes 4 and 5). Dephosphorylated CDK9 was resolved on 10% SDS-PAGE and quantified on PhosphoImager (lower panel). ***B***, Phosphorylase-*a *phosphatase activity of PP1 and PP2A at concentrations corresponding to panel A, presented as the amount of phosphorylase-*a *remained in the reaction after the treatment with the phosphatase. ***C***, Pre-treatment with PP2A increases autophosphorylation of CDK9. Recombinant CDK9/cyclin T1 was incubated without (lane 1) or with PP1 (lanes 2 and 3) or PP2A (lanes 4 and 5) at concentrations corresponding to Panel A. After incubation, the phosphatases were blocked with 1 μM okadaic acid and CDK9/cyclin T1 was subjected to the autophosphorylation with γ-(^32^P) ATP (lanes 1 to 5). Phosphorylated CDK9 was resolved on 10% SDS-PAGE and exposed to the PhosphoImager screen.

We explored whether PP2A or PP1 dephosphorylates CDK9 *in vitro*. CDK9 within the recombinant CDK9/cyclin T1 was autophosphorylated in the presence of γ-(P^32^)-ATP. The kinase activity of CDK9 was blocked by the addition of 7 mM EDTA and (^32^P) phosphorylated CDK9 was used as a substrate for PP1 or PP2A (Fig. 1A, lane 1). While PP2A efficiently dephosphorylated CDK9 (Fig. 1A, lanes 4 and 5), PP1 was approximately 10-time less efficient than PP2A in the dephosphorylation (Fig. 1A, lanes 2 and 3). Based on their activities towards the reference substrate, glycogen phosphorylase-a [27], PP1 was added at 1.5-fold higher activity than PP2A (Fig. 1B) and thus PP1 was even less efficient than PP2A, at least 20-time less efficient in dephosphorylation of CDK9.

### Dephosphorylation by PP2A enhances CDK9 autophosphorylation in vitro

**Figure 2:**
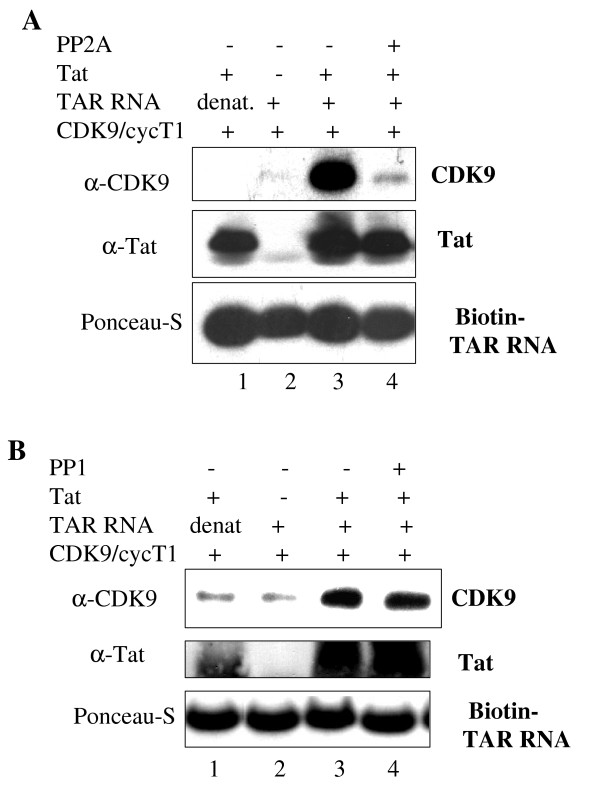
**Binding of Tat to TAR RNA and CDK9/cyclin T1**. Precipitation of biotin TAR RNA with purified Tat and with CDK9/cyclin T1. Lane 1, control denatured TAR RNA. Lane, control without Tat. Lane 3, untreated CDK9. cyclin T1. Lane 4, CDK9/cyclin T1 treated with PP2A (panel A) or with PP1 (panel B). Precipitated proteins and TAR RNA were recovered in SDS-loading buffer, resolved 12% SDS-PAGE and immunoblotted with indicated antibodies. Position of TAR RNA was determined by Ponceau-S staining.

Recently, CDK9 within the recombinant P-TEFb purified from insect cells was found to be phosphorylated on T186 [28]. We explore here whether CDK9 might be already in the phosphorylated state in our preparation of the recombinant P-TEFb. We asked whether dephosphorylation by either PP2A or PP1 of CDK9/cyclin T1 would enhance phosphorylation of CDK9 in the following kinase reaction. Recombinant CDK9/cyclin T1 was incubated with increasing concentrations of PP1 or PP2A followed by inhibition of the phosphatases with 1 μM okadaic acid. Then the autophosphorylation reaction was carried out in the presence of γ-(^32^P)-ATP (Fig. 1C). Treatment with PP2A (Fig. 1C, lanes 4 and 5) but not with PP1 (Fig. 2, lanes 2 and 3) increased the efficiency of CDK9 autophosphorylation. This result indicates that recombinant CDK9 was already in partially phosphorylated state and that PP2A-mediated dephosphorylation of CDK9 enhanced subsequent phosphorylation of CDK9.

### Dephosphorylation of CDK9 by PP2A prevents formation of P-TEFb/Tat/TAR RNA complex in vitro

We next analyzed whether dephosphorylation of CDK9 by PP2A or by PP1 has an effect on formation of a complex between recombinant P-TEFb, HIV-1 Tat and TAR RNA. We utilized a biotinylated TAR RNA that was preincubated with recombinant Tat and recombinant CDK9/cyclin T1 and then precipitated with streptavidin agarose beads (Figs. 2A and 2B, lane 3). When TAR RNA was denatured or when Tat was omitted, CDK9/cyclin T1 was not precipitated with TAR RNA (Figs. 2A and 2B, lanes 1 and 2) indicating a specific P-TEFb:Tat:TAR RNA complex formation. Pre-treatment of CDK9/cyclin T1 with PP2A resulted in a significant decrease in the complex formation (Fig. 2A, lane 4). In contrast, pretreatment of CDK9/cyclin T1 with PP1 did not have an effect on P-TEFb: Tat: TAR RNA complex formation (Fig. 2B, lane 4). These results indicate that PP2A but not PP1 affects formation of the P-TEFb: Tat: TAR RNA complex *in vitro*.

### Inhibition of PP2A by okadaic acid blocks basal and Tat-dependent HIV-1 transcription in vitro

**Figure 3:**
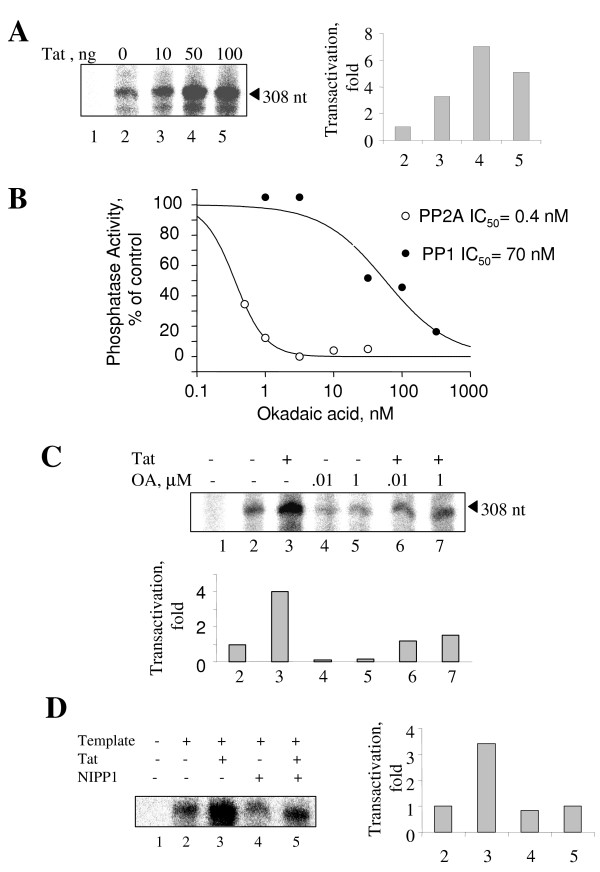
**Contribution of PP2A and PP1 to Tat-activated transcription *in vitro***. ***A***, *In vitro *transcription reactions were carried with the indicated amounts of recombinant Tat. Lane 1, no DNA template; lane 2, no Tat added; lanes 3–5, Tat added at 10 ng, 50 ng and 100 ng correspondingly. Transcription product was resolved on 5 % Urea-PAGE, exposed to the PhosphoImager screen and quantified. ***B***, Inhibition of PP1 and PP2A by okadaic acid in phosphorylase-*a *dephosphorylation assay. PP1 and PP2A were inhibited by okadaic acid with IC_50 _= 70 nM and 0.4 nM concentration of inhibitor respectively. ***C***, Okadaic acid inhibits basal and Tat-activated transcription. Lane 1, no DNA template; lane 2, no Tat added; lane 3, transcription with 50 ng of Tat; lanes 4 and 5, transcription in the absence of Tat and with 10 nM or 1 μM of okadaic acid; and lanes 6 and 7, transcription in the presence of 50 ng of Tat and with 10 nM or 1 μM of okadaic acid. Transcription products were resolved on 5 % Urea-PAGE, exposed to the PhosphoImager screen and quantified. ***D***, NIPP1 inhibits Tat-activated transcription. Lane 1, no DNA template; lane 2, no Tat added; lane 3, transcription with 50 ng of Tat; lane 4, transcription in the absence of Tat and with 100 ng NIPP1; lane 5, transcription in the presence of 50 ng of Tat and 100 ng NIPP1. Transcription products were resolved on 5 % Urea-PAGE, exposed to the PhosphoImager screen and quantified.

Next we analyzed whether inhibition of PP2A has an effect on HIV-1 transcription *in vitro*. An HIV-1 LTR template that contains 308 nucleotides downstream of the transcription start was prepared by PCR using HIV-1 LTR-LacZ expression vector (see Methods). Purified Tat stimulated transcription on this template in the HeLa nuclear extract to approximately 5-fold (Fig. 3A). We used okadaic acid, which is a 100-fold more efficient *in vitro *inhibitor of PP2A than PP1 (Fig. 3B) to determine the effect of PP2A inhibition on HIV-1 transcription. Two different concentrations of okadaic acid were used: 10 nM – to inhibit PP2A and 1 μM – to inhibit PP1. Addition of either 10 nM or 1 μM concentrations of okadaic acid inhibited basal HIV-1 transcription (Fig. 3C, compare lanes 4 and 5 to lane 2) and also Tat-activated HIV-1 transcription (Fig. 3C, compare lanes 6 and 7 to lane 3). Thus this result indicates that inhibition of PP2A blocks both basal and Tat-activated transcription.

### Inhibition of PP1 by NIPP1 blocks Tat-dependent HIV-1 transcription in vitro

we cannot rule out the possibility that PP1 might also be involved in the HIV-1 transcription *in vitro*. We used recombinant NIPP1 protein which we previously used to inhibit PP1 *in vitro *[29]. Similar to the experiment in the previous section, purified Tat stimulated transcription about 4-fold (Fig. 3D, compare lanes 2 and 3). Addition of NIPP1 inhibited Tat-activated transcription (Fig. 3D, lane 5), but did not affect basal HIV-1 transcription (Fig. 3D, lanes 4). This result indicates that PP1 might be involved in the Tat-activated transcription.

### Inhibition of PP1 but not PP2A significantly inhibits Tat-dependent HIV-1 transcription in cultured cells

**Figure 4:**
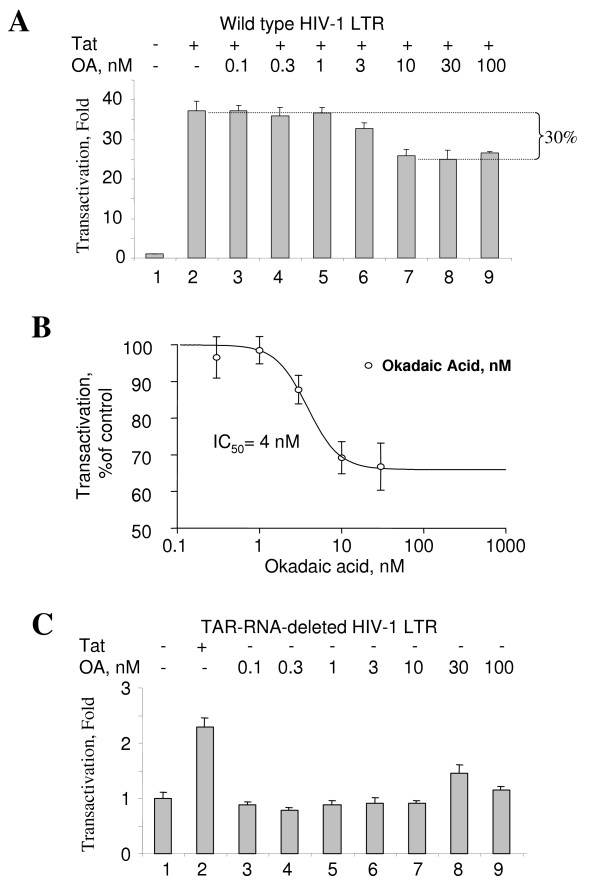
**Okadaic acid modestly inhibits Tat-induced HIV-1 transcription in cultured cells**. ***A***, COS-7 cells were co-transfected without (lane 1) or with Tat-expressing vector and HIV-1 LTR-LacZ (lanes 2–10). Cells were also treated with indicated concentrations of okadaic acid (lanes 3–10). Expression of β-galactosidase was analyzed using ONPG-based assay. ***B***, Quantification of the inhibition of Tat-induced transcription by okadaic acid using Prism. ***C***, COS-7 cells were transfected with mutant HIV-1 LTR with a deletion of the fragment encoding TAR RNA (HIV-1 LTRΔTAR) without (lanes 1 and 3–10) or with Tat-expression plasmid (lane 2) and treated with the indicated concentrations of okadaic acid (lanes 3–10).

**Figure 5:**
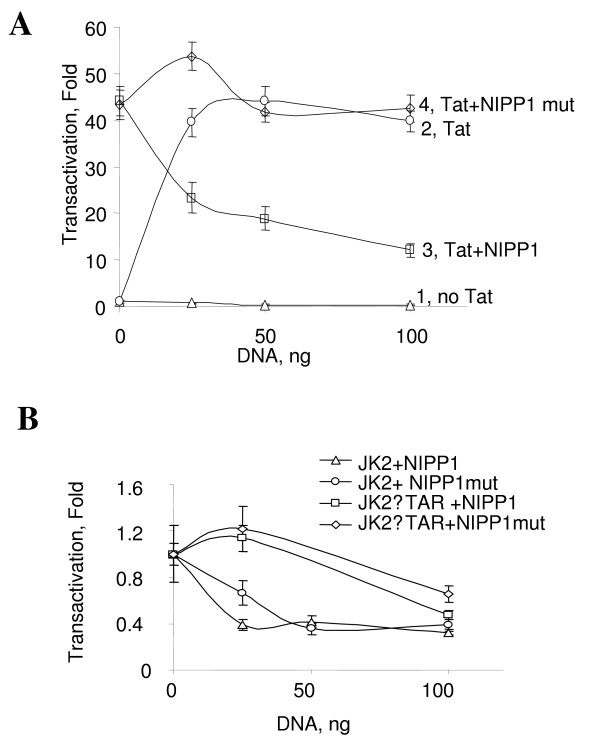
**Expression of NIPP1 inhibits Tat-dependent HIV-1 transcription in COS-7 cells**. ***A***, Lane 1, COS-7 cells grown in 24-well plate were transfected with the indicated amount of JK2 using Ca^2+^-phosphatase method. Lane 2, COS-7 cells were transfected with 25 ng of JK2 and indicated amount of Tat expression plasmid. Lane 3 and 4, COS-7 cells were transfected with 25 ng of JK2, 50 ng of Tat expression vector and indicated amounts of wild type or mutant NIPP1. ***B***, NIPP1 and mutant NIPP1 equally affect HIV-1 transcription in the absence of Tat. COS-7 cells were transfected with 50 ng of JK2 or JK2ΔTAR and with indicated amounts of NIPP1 or mutant NIPP1.

We next determined relative contribution of PP1 and PP2A to basal and Tat-activated HIV-1 transcription in cultured COS-7 cells using selective inhibition of PP2A and PP1. We used okadaic acid which selectively inhibits PP2A *in vitro *at concentrations below 1 nM (Fig. 3B) but which would inhibit both PP1 and PP2A at higher concentrations. COS-7 cells were co-transfected with Tat-expressing vector and HIV-1 LTR-LacZ (JK2) and expression of β-galactosidase was analyzed using quantitative ONPG-based assay [26]. In these cells Tat potently stimulate transcription from HIV-1 LTR (Fig. 4A, compare lanes 1 and 2). Treatment of the transfected COS-7 cells with okadaic acid resulted in partial (about 30%) inhibition of Tat-induced transcription (Fig. 4A). The IC_50 _of the okadaic acid-mediated inhibition was 4 nM (Fig. 4B). Surprisingly, okadaic acid had no inhibitory effect on HIV-1 basal transcription from a mutant HIV-1 LTR with a deletion of the fragment encoding TAR RNA (HIV-1 LTRΔTAR) (Fig. 4C). At the concentrations below 10 nM, treatment with okadaic acid did not affect viability of COS-7 cells (see supplemental Fig). These results indicate that PP2A has a moderate effect only on Tat-induced transcription in cultured cells. To analyze the contribution of PP1 to the control of HIV-1 transcription in COS-7 cells, vectors expressing NIPP1-EGFP WT or NIPP1-EGFP mutant (NIPP1 K193-197A/V201A/F203A/Y335D, NIPP1 mut) were transfected along with JK2 and Tat expression vector, as we previously described [26]. In the mutant NIPP1 the PP1 binding sites in both the central and C-terminal domain of NIPP1 are mutated and it no longer interacts with PP1 [30]. Co-transfection of wild type, but not the mutant NIPP1-EGFP, inhibited Tat-activated transcription (Fig. 5A, lanes 3 and 4). In the absence of Tat, transcription from HIV-1 LTR or from a mutant HIV-1 LTR with a deletion of the fragment encoding TAR RNA (JK2ΔTAR) was inhibited about 50% by both NIPP1 and mutant NIPP1 (Fig. 5B), indicating that this effect of NIPP1 was not due to its ability to bind PP1. Taken together, inhibition of PP1 by over expression of NIPP1 reduces Tat-dependent HIV-1 transcription by 70%, in accord to our previous study [26], Thus PP1 and less likely PP2A might contribute to the regulation of Tat-dependent HIV-1 transcription.

### CDK9 is dephosphorylated by PP1 in cultured cell

**Figure 6:**
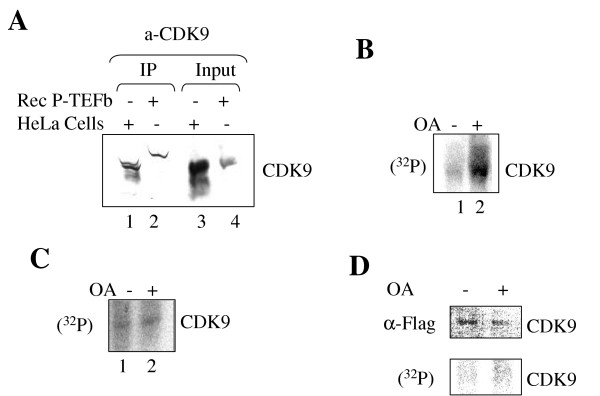
**CDK9 is dephosphorylated by PP1 in cultured cells**. ***A***, Immunoprecipitation of CDK9. Lane 1, CDK9 was precipitated from HeLa cell extract with anti-cyclin T1 antibodies, resolved on 10% SDS-PAGE and immunoblotted with anti-CDK9 antibodies; Lane 2, immunoprecipitation of recombinant CDK9/cyclin T1; Lanes 3, input recombinant CDK9/cyclin T1; lane 4, input HeLa cell extract. ***B***, HeLa cells were labelled with (^32^P) orthophosphate in the absence (lane 1) and in the presence of 1 μM okadaic acid (lane 2) and cellular extracts were immunoprecipitated with anti-cyclin T1 antibodies, resolved by 10% SDS-PAGE and transferred to PVDF membrane. Position of CDK9 was determined by probing the membrane with anti-CDK9 antibodies using 3,3'-Diaminobenzidine enhancer system. The picture is autoradiogram of the membrane exposed to phosphor imager screen. ***C***, 293T cells were labeled with (^32^P) orthophosphate in the absence (lane 1) and in the presence of 100 nM okadaic acid (lane 2) and cellular extracts were immunoprecipitated with anti-CDK9 antibodies, resolved by 10% SDS-PAGE and transferred to PVDF membrane. Position of CDK9 was determined by probing the membrane with anti-CDK9 antibodies using 3,3'-Diaminobenzidine enhancer system. The picture is an autoradiogram of the membrane exposed to phosphor imager screen. ***D***, 293T-cdNIPP1 cells stably expressing central domain of NIPP1 (143–224) were transfected with Flag-CDK9 expression vector and labeled with (^32^P) orthophosphate in the absence (lane 1) and in the presence of 100 nM okadaic acid (lane 2). Cellular extracts were immunoprecipitated with anti-Flag antibodies, resolved by 10% SDS-PAGE and transferred to PVDF membrane. Position of CDK9 was determined by probing the membrane with anti-CDK9 antibodies using 3,3'-Diaminobenzidine enhancer system. The picture is autoradiogram of the membrane exposed to phosphor imager screen.

We next analyzed whether CDK9 phosphorylation state is controlled by PP1 or by PP2A in cultured cells. HeLa cells were labelled with (^32^P) orthophosphate in the absence and in the presence of okadaic acid and cellular extracts were immunoprecipitated with anti-cyclin T1 antibodies, resolved by 10% SDS-PAGE and transferred to PVDF membrane. Position of CDK9 was determined by probing the membrane with anti-CDK9 antibodies using 3,3'-Diaminobenzidine enhancer system (Fig. 6A). Precipitation of CDK9 from untreated cells and from the cells treated with okadaic acid showed that phosphorylation of CDK9 was increased in the presence of okadaic acid (Fig. 6B, lanes 1 and 2). To analyze whether this increase was due to inhibition of PP1 or PP2A, we utilized 293T cells that were stably transfected with the central domain of NIPP1 (residues 143–224) (293T-cdNIPP1 cells), an equally potent inhibitor of PP1 as full length NIPP1 [30]. Precipitation of endogenous CDK9 from untreated 293T-cdNIPP1 cells labeled with (^32^P) in the absence or in the presence of 100 nM okadaic acid showed equal low level of CDK9 phosphorylation (Fig. 6C, lanes 1 and 2). To further investigate whether CDK9 phosphorylation was caused by PP1, we transiently express Flag- tagged CDK9 in 293T-cdNIPP1 cells, labeled cells with (^32^P) and precipitated CDK9 with anti-Flag antibodies. Again treatment with 100 nM okadaic acid did not increase phosphorylation of CDK9 (Fig. 6D, lanes 1 and 2). Taken together these results indicate that CDK9 is dephosphorylated *in vivo *and that it is likely PP1 that dephosphorylates CDK9.

**Figure 7:**
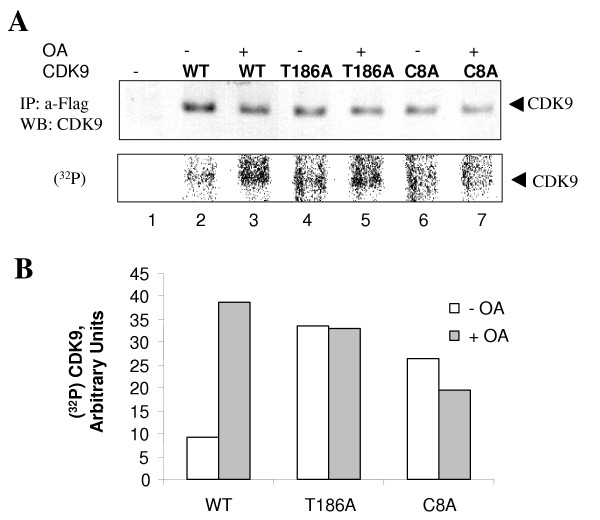
**Determination of CDK9 residues dephosphorylated in cultured cells**. ***A***, 293T cells were transfected with expression vectors Flag-tagged CDK9 (lanes 2 and 3), CDK9 T186A mutant (lanes 4 and 5) and CDK9 C8A mutant (lanes 6 and 7). Cells were labeled with (^32^P) orthophosphate in the absence (lanes 2, 4 and 6) and in the presence of 100 nM okadaic acid (lane 3, 5 and 7). Lane 1, mock transfected cells. Cellular extracts were immunoprecipitated with anti-Flag antibodies, resolved by 10% SDS-PAGE and transferred to PVDF membrane. Upper panel shows expression of CDK9 determined by probing the membrane with anti-CDK9 antibodies using 3,3'-Diaminobenzidine enhancer system. Lower panel is an autoradiogram of the membrane exposed to phosphor imager screen. ***B***, quantification of the Phosphor Imager panel.

To further explore the CDK9 dephosphorylation, we analyzed phosphorylation of CDK9 mutants with mutation in Thr 186 (T186A mutant) or with mutations in Ser-329, Thr-330, Thr-333, Ser-334, Ser-347, Thr-350, Ser-353, and Thr-354 residues (C8A mutant). 293T cells were transiently transfected with Flag-tagged CDK9, WT, T186A mutant or C8A mutant. Transfected cells were labeled with (^32^P) without or with the addition of 100 nM okadaic acid. Precipitation of CDK9 with anti-Flag antibodies showed that while okadaic acid induced phosphorylation of WT CDK9 (Fig. 7A, lanes 2 and 3; and Fig. 7B), there was no further increase in phosphorylation of T186A or C8A mutant (Fig. 7A, lanes 4 to 7: and Fig. 7B). Interestingly, mutation of Thr 186 increases CDK9 phosphorylation level (Fig. 7A, lane 5 and Fig. 7B).

Taken together our results indicate that PP1 may potentially dephosphorylate Thr 186 as well as the C-terminal serines involved in the autophosphorylation of CDK9 and that dephosphorylation of CDK9 may have a regulatory effect in Tat-activated HIV-1 transcription.

## Discussion

In this study, we show that while PP2A dephosphorylates CDK9 *in vitro*, in cultured cells PP1 preferentially dephosphorylates CDK9 and largely contributes to the regulation of activated HIV-1 transcription. Previously PP2A has been shown to stimulate HIV-1 transcription [22]. Because PP2A exists in multiply complexes it is still not clear what is the substrate for PP2A during HIV-1 transcription. Results presented in the present paper show that it is unlikely that PP2A dephosphorylates CDK9 *in vivo*. Previously, CDK9 phosphorylation was linked to the binding of CDK9/cyclin T1 to TAR RNA in the presence of Tat [18]. Our *in vitro *data are clearly in agreement with the earlier observations. Recently, acetylation of the RNA binding region of Tat was shown to be important for Tat function *in vivo *and it was proposed to help in dissociating CyclinT1 from TAR RNA [31]. Thus it is remained to be determined whether autophosphorylation of CDK9 is important for P-TEFb interaction with TAR RNA *in vivo *and whetherphosphorylation of the C-terminus of CDK9 is linked to the acetylation of Tat. In our study, PP2A affected both basal and Tat-induced HIV-1 transcription. This indicates that PP2A may be important for the early steps of transcription. Since β-galactosidase is quite stable, our experimental system allows us to measure only general cumulative effects and thus we may have overlooked the early transcriptional effects. The inhibitory effect of NIPP1 on Tat-dependent transcription *in vitro *agrees well with our previous observation that inhibition of PP1 blocks Tat-activated but not basal HIV-1 transcription [26]. But generally the effect of Tat *in vitro *in our system was relatively small, only 3–5 folds induction, as compared to the 30-fold or more induction in the cells. Thus it is possible that either the basal transcription *in vitro *was artificially high, or that the Tat activation only partially reproduces the situation *in vivo*. Our unpublished observations indicate that Tat may directly interact with PP1 *in vivo *and retarget PP1 within the cells, the effect that may not be seen *in vitro*. We chose for the analysis COS-7 cells in which HIV-1 transcription is not induced in response to the low concentration of the okadaic acid, likely because of the retargeting of PP2A by SV40 small T antigen [32]. We showed that low concentrations of okadaic acid (IC_50 _= 4 nM) mildly inhibit Tat-induced but not the basal HIV-1 transcription. The level of the achieved inhibition was only 30% indicating that phosphatases, including PP1 may also contribute to the regulation of HIV-1 transcription. Our previous study [26] and the results presented here indicate that PP1 may be one of the candidate phosphate, as inhibition of nuclear PP1 potently blocked Tat-transactivation. Analysis of the CDK9 phosphorylation in cultured cells showed that its phosphorylation is likely to be controlled by PP1 because in the cells, that stably express central domain of NIPP1, there was no increase of CDK9 phosphorylation in the presence of okadaic acid. A more complex explanation is that PP1 might regulate PP2A activity and thus indirectly affect CDK9 phosphorylation. Although the moderate inhibitory effect of okadaic acid on HIV-1 transcription argues against this possibility, we cannot exclude it completely. CDK9/cyclin T1 was shown to bind to its inhibitors, 7SK RNA and MAQ1/HEXIM1 protein in phosphorylation-dependent manner [33]. Autophosphorylation of CDK9 takes place in the C-terminus [18], whereas a yet unknown cellular kinase phosphorylates CDK9 within the regulatory T-loop [33]. It was proposed that phosphorylation of Thr 186 inhibits the activity of P-TEFb and that its dephosphorylation reactivates P-TEFb by allowing dissociation of 7SK RNA and HEXIM1 [33]. In a contradictory study, Price and colleagues showed that phosphorylation of Thr 186 is required for the kinase activity of CDK9 and argued against the regulatory role of dephosphorylation of Thr186 [28]. Our study points to a possibility to resolve this discrepancy by determining the phosphorylation state of Thr 186 and the activity of endogenous CDK9 in the cells which continuously express central domain of NIPP1. Taking together, our study demonstrates that PP1 is likely to dephosphorylate CDK9 *in vivo *and that inhibitory effect of NIPP1 on HIV-1 transcription might be due to the deregulation of CDK9 phosphorylation.

## Methods

### Materials

COS-7 cells, 293T cells and HeLa cells were purchased from ATCC (Manassas, VA). 293T cells stably expressing NIPP1 (143–224) were generated by transfection of NIPP1-143-224-EGFP, and limited dilution cloning in the presence of geneticin (0.5 mg/ml) (Life Technologies, Rockville, MD). Human protein phosphatase PP2A was purchased from Upstate Biotechnology (Lake Placid, NY). Rabbit protein phosphatase PP1 and recombinant NIPP1 were gifts from M. Bollen (Catholic University, Leuven, Belgium). Phosphorylase *b *was from Calzyme Laboratories (San Luis Obispo, CA). Protein (A) agarose was purchased from Sigma (Atlanta, GA). Human recombinant P-TEFb from baculovirus transfected Sf9 cells (NCCC) was purified as described in [34].

### Antibodies

Rabbit polyclonal antibodies to CDK9 and goat polyclonal antibodies to cyclin T1 were purchased from Santa Cruz Biochemical (Santa Cruz, CA).

### Plasmids

The reporter plasmid, pJK2, contained HIV-1 LTR (-138 to +82) followed by nuclear localization signal (NLS) and *lacZ *reporter gene (courtesy of Dr. Michael Emerman, Fred Hutchinson Cancer Institute, Seattle, WA). This plasmid expresses NLS-tagged β-galactosidase under the control of HIV-1 LTR [35]. Tat expression plasmid was a gift from Dr. Ben Berkhout (University of Amsterdam) [36]. The pGEM2Tat bacterial expression vector was obtained from NIH AIDS Research and Reference Reagents Program.

### CDK9 autophosphorylation and dephosphorylation in vitro

Recombinant CDK9/cyclin T1 (30 ng/reaction) was autophosphorylated in 20 μl reaction with 50 μM ATP (1 μCi of γ-(^32^P)ATP) in kinase buffer (50 mM HEPES (pH 7.9), 10 mM MgCl_2_, 6 mM EGTA and 2.5 mM DTT) for 1 hour at 30°C. The reaction was supplemented with 7 mM EDTA to inactivate the kinase, followed by addition of PP2A or PP1 and incubation for 30 min at 30°C. Dephosphorylation of recombinant CDK9/cyclin T1 was carried out in Tris-HCl buffer pH 8.0, 5 mM MgCl_2_, 5 mM MnCl_2_, 20 μM ZnSO_4 _using indicated amount of PP1 or PP2A. The phosphatases were inhibited with 1 μM okadaic acid. The 250 nM ATP and 5 μCi γ-(^32^P)ATP were added in the same buffer and incubated for 30 min at 30°C. Reactions were resolved on 10% SDS-PAGE and subjected to autoradiography and quantification with PhosphorImager Storm 860 (Molecular Dynamics).

### Preparation of phosphorylase-a and dephosphorylation assay

10 mg of phosphorylase-b was dissolved in 300 μl BFA (10 mM glycerophosphate pH 7.4, 50 mM 2-mercaptoethanol) and dialyzed against the same buffer for 2 to 3 hours. Then 7.5 μl 500 mM Tris-HCl (pH 8.0) and 6 μl phosphorylase kinase were added and incubated 10 min 30°C followed by addition of 45 μl ATP-Mg mix (8.3 mM ATP, 83 mM MgCl_2_, 75 μCi γ-(^32^P)ATP) and incubation for 2 h at 30°C. Phosphorylase-a was precipitated with ammonium sulfate, resuspended in BFA and dialyzed against BFA for 1 to 2 day at 4°C. AG 501 × 8 (mixed anion and cation exchange) resin was placed in a separate dialysis bag to improve removal of unincorporated ATP and inorganic phosphate. Dialyzed phosphorylase-a was kept at 4°C. Approximately 0.2 nmol of phosphorylase-a was used as a substrate for PP1 or PP2A. The phosphorylase phosphatase assay was carried out for 10 min in a buffer containing 50 mM glycylglycine at pH 7.4, 0.5 mM dithiothreitol, and 5 mM β-mercaptoethanol as described [27].

### In vitro interaction of biotinylated TAR RNA, Tat and CDK9/cyclin T1

Biotin-TAR RNA (51 nucleotides) was purchased from Molecula company . To bind TAR RNA, streptavidin-agarose beads were washed with binding buffer (20 mM Tris-HCl, pH 7.5, 2.5 mM MgCl_2_, 100 mM NaCl) and incubated with TAR RNA (5 μg/reaction) or formamide denatured TAR RNA for 30 min at 4°C. The beads were washed with the binding buffer and incubated with recombinant Tat protein (1.5 μg/reaction) for 30 min at 4°C. Beads were washed with binding buffer and then with TAK buffer (50 mM Tris-HCl, pH 8.0, 5 mM MgCl_2_, 5 mM MnCl_2_, 10 μM ZnSO_4_, 1 mM DTT) containing 100 mM NaCl. Then 64 μg of yeast tRNA and 100 μg of BSA were added per reaction to block the beads. Then recombinant CDK9/cyclin T1 (30 ng/reaction) was added in TAK buffer containing 100 mM NaCl. Protein phosphatases PP1 and PP2A were diluted in TAK buffer and added into reaction where indicated at 0.1U of PP1 or 0.04U of PP2A. Samples were incubated for 1 h on ice with occasional mixing. Then beads were washed 3 times with the binding buffer, and bound proteins and RNA were eluted in 1× SDS-loading buffer. Proteins were separated on 12% SDS-PAGE, transfer to PVDF membrane, immunoblotted with α-CDK9 (Santa Cruz Biotechnology) and α-Tat 4A4.8 (NIH, AIDS Research Program) antibodies. Biotin TAR RNA was detected by Ponceau S staining.

### In vitro transcription assay

Biotinylated HIV-1 LTR DNA template which included-111 to +308 nucleotides of JK2 (HIV-1 LTR nucleotides-111 to +82) was amplified by PCR with the forward primer 5' biotinylated-TTCTACAAGGGACTTTCCGC-3' and the reverse primer 5'-CAGTACAGGCAAAAAGCAGC-3' (Life Technologies, Rockville, MD). Transcription reactions (20 μl) contained 75 μg of HeLa nuclear extract, 0.5 mM of each ATP, CTP and GTP, 20 μM UTP, 2 μCi [α^32^P]UTP, 0.2–0.4 μg of the template in transcription buffer (20 mM HEPES at pH 7.9, 50 mM KCl, 6.25 mM MgCl_2_, 0.5 mM EDTA, 2 mM DTT and 10% glycerol), RNAsin and a recombinant pGEM2 Tat72 for transactivation reaction. 10 nM okadaic acid has been used for inhibition of serine/threonine phosphatases. After 30 min incubation at 30°C, reactions were treated with proteinase K for 15 min at 55°C, and extracted with phenol-chloroform-isoamylalcohol(LifeTechnologies, Rockville, MD). RNA was precipitated from the water phase and resolved on 5% sequencing polyacrylamide gels containing 7 M urea. Mcp 1 digested PBR 322 plasmid (LifeTechnologies, Rockville, MD) labeled with Klenow fragment and ^32^P-labeled dCTD served as molecular weight markers. The labeling was quantified by phosphor imaging (Packard Instruments).

### Transient transfections

COS-7 cells were cultured at 7 × 10^5 ^cells/well in DMEM containing 10% fetal bovine serum. Co-transfections with Tat-expressing vector and HIV-1 LTR-LacZ or HIV-1 LTRΔTAR were performed at 75% confluency using a Ca^2+ ^– phosphate protocol and the indicated reporter plasmids. After transfection the cells were cultured for an additional 48 hours and β-galactosidase activity was analyzed using quantitative ONPG-based assay [26]. Where indicated, okadaic acid was added to transfected cells. Transfections were normalized using MTT assay (Sigma).

### β-galactosidase assays

Cells were washed with phosphate-buffered saline (PBS) and lysed for 20 min at room temperature in 50 μl of lysis buffer, containing 20 mM HEPES at pH 7.9, 0.1% NP-40 and 5 mM EDTA. Subsequently, 100 μl of o-nitrophenyl-β-D-galactopyranoside (ONPG) solution (72 mM Na_2 _PO_4 _at pH 7.5, 1 mg/ml ONPG, 12 mM MgCl_2_, 180 mM 2-mercaptoethanol) was added and incubated at room temperature until a yellow color was developed. The reaction was stopped by addition of 100 μl of 1 M Na_2_CO_3_. The 96-well plate was analyzed in a micro plate reader at 414 nm (Lab Systems Multiscan MS).

### In vivo labeling with (^32^P) orthophosphate and Western blot

HeLa or 293T cells were incubated with phosphate-free DMEM media (Life Technologies, Rockville, MD) containing no serum for 1 hour. The media was changed to phosphate-free DMEM media supplemented with 0.5 mCi/ml of (^32^P)-orthophosphate and cells were further incubated for 2 hours at 37°C. Where indicated, 0.1 μM okadaic acid (Sigma) was added to block cellular PPP-phosphatases. Cells were washed with PBS and lyzed in a buffer containing 50 mM Tris-HCl, pH 7.5, 0.5 M NaCl, 1% NP-40, 0.1% SDS and protease cocktail (Sigma). After 10 min on ice, cellular material was scraped and then centrifuged at 14,000 rpm, 4°C for 30 min. The supernatant was recovered and used for immunoprecipitation. CDK9 was co-precipitated with anti-cyclin T1 antibodies coupled to protein A agarose for 2 h at 4°C in a TNN Buffer containing 50 mM Tris-HCl, pH 7.5, 0.15 M NaCl, and 1% NP-40. The immunoprecipitated P-TEFb was recovered by heating for 2 min at 100°C in Tris-SDS loading buffer, resolved on 10% SDS-PAGE (25) and transferred to polyvinylidene fluoride (PVDF) membranes (Millipore, Allen, TX). The membrane was analyzed with anti-CDK9 polyclonal antibodies using 3,3'-Diaminobenzidine enhancer system (Sigma) and was also exposed to Phosphor Imager screen (Packard Instruments, Wellesley, MA).

## Competing interests

The author(s) declare that they have no competing interests.

## Authors' contributions

TA carried out *in vitro *transcription studies, phosphorylase phosphatase assays and experiments on CDK9 phosphorylation in cultured cells. KW performed cell transfection experiments. ZB helped with the expression of Flag-tagged CDK9. JB participated in the design and discussion of the study and provided purified CDK9/cyclin T1. SN performed *in vitro *CDK9 dephosphorylation assays, performed general control and coordination of the study. All authors read and approved the manuscript.

## Supplementary Material

Additional File 1 **Supplemental Fig**. Viability of COS-7 cells treated with indicated concentrations of okadaic acid determined by Trypan Blue exclusion assay. Click here for file

## References

[B1] Marcello A, Zoppe M, Giacca M (2001). Multiple modes of transcriptional regulation by the HIV-1 Tat transactivator. IUBMB Life.

[B2] Liang C, Wainberg MA (2002). The role of Tat in HIV-1 replication: an activator and/or a suppressor?. AIDS Rev.

[B3] Giacca M (2004). The HIV-1 Tat protein: a multifaceted target for novel therapeutic opportunities. Curr Drug Targets Immune Endocr Metabol Disord.

[B4] Brigati C, Giacca M, Noonan DM, Albini A (2003). HIV Tat, its TARgets and the control of viral gene expression. FEMS Microbiol Lett.

[B5] Kim YK, Bourgeois CF, Isel C, Churcher MJ, Karn J (2002). Phosphorylation of the RNA polymerase II carboxyl-terminal domain by CDK9 is directly responsible for human immunodeficiency virus type 1 Tat-activated transcriptional elongation. Mol Cell Biol.

[B6] Zhu Y, Pe'ery T, Peng J, Ramanathan Y, Marshall N, Marshall T, Amendt B, Mathews MB, Price DH (1997). Transcription elongation factor P-TEFb is required for HIV-1 tat transactivation in vitro. Genes Dev.

[B7] Raha T, Cheng SW, Green MR (2005). HIV-1 Tat stimulates transcription complex assembly through recruitment of TBP in the absence of TAFs. PLoS Biol.

[B8] Jeang KT, Berkhout B, Dropulic B (1993). Effects of integration and replication on transcription of the HIV-1 long terminal repeat. J Biol Chem.

[B9] Jeang KT, Berkhout B (1992). Kinetics of HIV-1 long terminal repeat trans-activation. Use of intragenic ribozyme to assess rate-limiting steps. J Biol Chem.

[B10] Kashanchi F, Piras G, Radonovich MF, Duvall JF, Fattaey A, Chiang CM, Roeder RG, Brady JN (1994). Direct interaction of human TFIID with the HIV-1 transactivator tat. Nature.

[B11] Herrmann CH, Rice AP (1995). Lentivirus Tat proteins specifically associate with a cellular protein kinase, TAK, that hyperphosphorylates the carboxyl-terminal domain of the large subunit of RNA polymerase II: candidate for a Tat cofactor. J Virol.

[B12] Yang X, Gold MO, Tang DN, Lewis DE, Aguilar-Cordova E, Rice AP, Herrmann CH (1997). TAK, an HIV Tat-associated kinase, is a member of the cyclin-dependent family of protein kinases and is induced by activation of peripheral blood lymphocytes and differentiation of promonocytic cell lines. Proc Natl Acad Sci U S A.

[B13] Kiernan RE, Vanhulle C, Schiltz L, Adam E, Xiao H, Maudoux F, Calomme C, Burny A, Nakatani Y, Jeang KT, Benkirane M, Van Lint C (1999). HIV-1 tat transcriptional activity is regulated by acetylation. Embo J.

[B14] Ott M, Schnolzer M, Garnica J, Fischle W, Emiliani S, Rackwitz HR, Verdin E (1999). Acetylation of the HIV-1 Tat protein by p300 is important for its transcriptional activity. Curr Biol.

[B15] Deng L, de la Fuente C, Fu P, Wang L, Donnelly R, Wade JD, Lambert P, Li H, Lee CG, Kashanchi F (2000). Acetylation of HIV-1 Tat by CBP/P300 increases transcription of integrated HIV-1 genome and enhances binding to core histones. Virology.

[B16] Bieniasz PD, Grdina TA, Bogerd HP, Cullen BR (1998). Recruitment of a protein complex containing Tat and cyclin T1 to TAR governs the species specificity of HIV-1 Tat. Embo J.

[B17] Garber ME, Wei P, KewalRamani VN, Mayall TP, Herrmann CH, Rice AP, Littman DR, Jones KA (1998). The interaction between HIV-1 Tat and human cyclin T1 requires zinc and a critical cysteine residue that is not conserved in the murine CycT1 protein. Genes Dev.

[B18] Garber ME, Mayall TP, Suess EM, Meisenhelder J, Thompson NE, Jones KA (2000). CDK9 autophosphorylation regulates high-affinity binding of the human immunodeficiency virus type 1 tat-P-TEFb complex to TAR RNA. Mol Cell Biol.

[B19] Zhou M, Nekhai S, Bharucha DC, Kumar A, Ge H, Price DH, Egly JM, Brady JN (2001). TFIIH inhibits CDK9 phosphorylation during human immunodeficiency virus type 1 transcription. J Biol Chem.

[B20] Bollen M, Beullens M (2002). Signaling by protein phosphatases in the nucleus. Trends Cell Biol.

[B21] Ruediger R, Brewis N, Ohst K, Walter G (1997). Increasing the ratio of PP2A core enzyme to holoenzyme inhibits Tat-stimulated HIV-1 transcription and virus production. Virology.

[B22] Faulkner NE, Lane BR, Bock PJ, Markovitz DM (2003). Protein phosphatase 2A enhances activation of human immunodeficiency virus type 1 by phorbol myristate acetate. J Virol.

[B23] Beullens M, Van Eynde A, Stalmans W, Bollen M (1992). The isolation of novel inhibitory polypeptides of protein phosphatase 1 from bovine thymus nuclei. J Biol Chem.

[B24] Jagiello I, Beullens M, Stalmans W, Bollen M (1995). Subunit structure and regulation of protein phosphatase-1 in rat liver nuclei. J Biol Chem.

[B25] Bharucha DC, Zhou M, Nekhai S, Brady JN, Shukla RR, Kumar A (2002). A protein phosphatase from human T cells augments tat transactivation of the human immunodeficiency virus type 1 long-terminal repeat. Virology.

[B26] Ammosova T, Jerebtsova M, Beullens M, Voloshin Y, Ray PE, Kumar A, Bollen M, Nekhai S (2003). Nuclear protein phosphatase-1 regulates HIV-1 transcription. J Biol Chem.

[B27] Beullens M, Stalmans W, Bollen M (1998). The biochemical identification and characterization of new species of protein phosphatase 1. Methods Mol Biol.

[B28] Li Q, Price JP, Byers SA, Cheng D, Peng J, Price DH (2005). Analysis of the large inactive P-TEFb complex indicates that it contains one 7SK molecule, a dimer of HEXIM1 or HEXIM2, and two P-TEFb molecules containing Cdk9 phosphorylated at threonine 186. J Biol Chem.

[B29] Washington K, Ammosova T, Beullens M, Jerebtsova M, Kumar A, Bollen M, Nekhai S (2002). Protein phosphatase-1 dephosphorylates the C-terminal domain of RNA polymerase-II. J Biol Chem.

[B30] Beullens M, Vulsteke V, Van Eynde A, Jagiello I, Stalmans W, Bollen M (2000). The C-terminus of NIPP1 (nuclear inhibitor of protein phosphatase-1) contains a novel binding site for protein phosphatase-1 that is controlled by tyrosine phosphorylation and RNA binding. Biochem J.

[B31] Kaehlcke K, Dorr A, Hetzer-Egger C, Kiermer V, Henklein P, Schnoelzer M, Loret E, Cole PA, Verdin E, Ott M (2003). Acetylation of Tat defines a cyclinT1-independent step in HIV transactivation. Mol Cell.

[B32] Moreno CS, Ramachandran S, Ashby DG, Laycock N, Plattner CA, Chen W, Hahn WC, Pallas DC (2004). Signaling and transcriptional changes critical for transformation of human cells by simian virus 40 small tumor antigen or protein phosphatase 2A B56gamma knockdown. Cancer Res.

[B33] Chen R, Yang Z, Zhou Q (2004). Phosphorylated positive transcription elongation factor b (P-TEFb) is tagged for inhibition through association with 7SK snRNA. J Biol Chem.

[B34] Peng J, Zhu Y, Milton JT, Price DH (1998). Identification of multiple cyclin subunits of human P-TEFb. Genes Dev.

[B35] Kimpton J, Emerman M (1992). Detection of replication-competent and pseudotyped human immunodeficiency virus with a sensitive cell line on the basis of activation of an integrated beta-galactosidase gene. J Virol.

[B36] Verhoef K, Bauer M, Meyerhans A, Berkhout B (1998). On the role of the second coding exon of the HIV-1 Tat protein in virus replication and MHC class I downregulation. AIDS Res Hum Retroviruses.

