# Supplementary figures and images for: Dephosphorylation of CDK9 by protein phosphatase 2A and protein phosphatase-1 in Tat-activated HIV-1 transcription
Source: Retrovirology. 2005 Jul 27;2:47. doi: 10.1186/1742-4690-2-47 (PMC1187922; doi:10.1186/1742-4690-2-47)

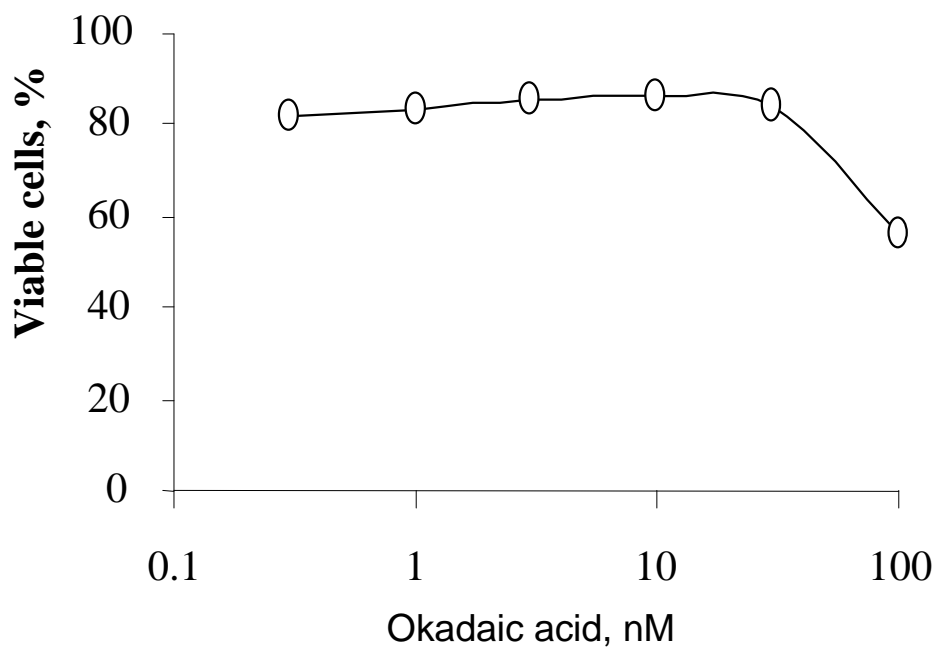

Supplement: Additional File 1 — Supplemental Fig. Viability of COS-7 cells treated with indicated concentrations of okadaic acid determined by Trypan Blue exclusion assay. [file 1742-4690-2-47-S1.pdf]
